# HPV infection and number of lifetime sexual partners are strong predictors for ‘natural’ regression of CIN 2 and 3

**DOI:** 10.1038/sj.bjc.6601196

**Published:** 2003-09-09

**Authors:** J K Chan, B J Monk, C Brewer, K A Keefe, K Osann, S McMeekin, G S Rose, M Youssef, S P Wilczynski, F L Meyskens, M L Berman

**Affiliations:** 1 Division of Gynecology Oncology, Department of Obstetrics and Gynecology, Stanford University Medical Center, 300 Pasteur Drive, Stanford, CA 94305, USA; 2 Division of Gynecologic Oncology, Department of Obstetrics and Gynecology, Chao Family Comprehensive Cancer Center, University of California, Irvine Medical Center, 101 The City Drive, Orange, CA 92868, USA; 3 Division of Hematology/Oncology, Department of Medicine, Chao Family Comprehensive Cancer Center, University of California, Irvine Medical Center, 101 The City Drive, Orange, CA 92868, USA; 4 Department of Pathology, The City of Hope National Medical Center, 1500 East Duarte Road, Duarte, CA 91010, USA

**Keywords:** high-grade cervical dysplasia, predictors, natural regression

## Abstract

The aim of this paper was to evaluate the factors that predict regression of untreated CIN 2 and 3. A total of 93 patients with colposcopic persistent CIN 2 and 3 lesions after biopsy were followed for 6 months. Human papillomavirus (HPV) types were determined by polymerase chain reaction at enrolment. We analysed the biologic and demographic predictors of natural regression using univariate and multivariate methods. The overall regression rate was 52% (48 out of 93), including 58% (22 out of 38) of CIN 2 and 47% (26 out of 55) of CIN 3 lesions (*P*=0.31 for difference). Human papillomavirus was detected in 84% (78 out of 93) of patients. In univariate analysis, 80% (12 out of 15) of lesions without HPV regressed compared to 46% (36 out of 78) of lesions with HPV infection (*P*=0.016). Women without HPV and those who had a resolution of HPV had a four-fold higher chance of regression than those with persistent HPV (relative odds=3.5, 95% CI=1.4–8.6). Women with five or fewer lifetime sexual partners had higher rates of regression than women with more than five partners (*P*=0.003). In multivariate analysis, HPV status and number of sexual partners remained as significant independent predictors of regression. In conclusion, HPV status and number of lifetime sexual partners were strongly predictive of regression of untreated CIN 2 and 3.

Cervical cancer continues to be the most deadly female cancer in developing countries. (Ponten *et al*, 1995) The dramatic decrease in mortality rates in Western countries is attributed to cervical cancer screening. (Harlan *et al*, 1991) The successful implementation of these screening programmes is based on the underlying model that cervical intraepithelial lesions (CIN) behave as progressive stages of a biologic continuum towards the development of invasive cervical cancer (Richart and Barron, 1969; Campion *et al*, 1986).

Genital human papillomavirus (HPV) infection has been established as the critical step in the development of most cervical cancers (IARC, 1995). The cervical carcinogenesis model involves HPV infection, intraepithelial neoplasia, and invasion. Human papillomavirus infection is found in over 40% of young, sexually active, college students (Ho *et al*, 1998). However, the incidence of cervical cancer is uncommon compared to the lifetime cumulative incidence of viral infection. Thus, there must be essential biological mechanisms and associated risk factors other than HPV infection that are responsible for the progression of precursor lesions to invasive cervical cancer (Schiffman & Brinton, 1995).

The aetiology and risk factors involved with cervical carcinogenesis are amenable to study as invasive cervical cancer typically arises after many years from a morphologically defined precancerous lesion. However, since untreated high-grade lesions can progress to invasive cancer, most women with CIN 2 or 3 are treated promptly. Accordingly, there are few reports on the natural history of high-grade dysplasia. On the other hand, not all CIN lesions progress to invasive cancer. In fact, spontaneous regression of moderate dysplasia has been reported in up to 54% of women (Nasiell *et al*, 1983, 1986). Since many early lesions regress without treatment, the ability to predict which lesions will regress offers the hope of avoiding treatment and its associated complications which include: cervical stenosis, incompetence, bleeding, and infection (Hillard *et al*, 1991; Herzog *et al*, 1995).

We evaluated the predictors for natural regression of untreated CIN 2 and 3 such as age, race, parity, smoking history, oral contraceptive (OCP) use, marital status, lesion grade, sexual history, and HPV status on all our patients.

## MATERIALS AND METHODS

We performed an ancillary study to a prospective, double-blinded, randomized, placebo-controlled trial of *β*-carotene in the management of women with untreated CIN 2 or 3. Predictors for natural regression, which included age, race, parity, smoking history, OCP use, marital status, lesion grade, sexual history, and HPV status were prospectively analysed. Patients were recruited from the University of California, Irvine, Medical Center, nearby clinics, and colleges. Women greater than 17 years of age with biopsy-proven CIN 2 and 3 were screened. Eligible patients had colposcopic persistence of CIN 2 or 3 one month after initial biopsy and had a negative endocervical curettage. Two pathologists agreed on the cervical histologic diagnosis before enrolment. Based on the guidelines of the Institutional Review Board at University of California, Irvine, written consents were obtained from all patients.

Since many preinvasive changes can regress spontaneously, we were initially interested in studying the factors that influenced the natural regression of CIN in the placebo group of our trial. However, we discovered that there was no significant difference in regression rates between the *β*-carotene and placebo arms at the end of the study (Keefe *et al*, 2001). Thus, we elected to look at those factors that influenced CIN regression in all study participants assuming that all patients who regressed did so spontaneously.

### Diagnosis of cervical dysplasia and surveillance

This was a 6-month ancillary study of a 2-year randomised, controlled trial of *β*-carotene in the management of women with untreated CIN 2 or 3. Over the 6 months, which comprised the study period for this current report, the subjects were followed at 3-month intervals with testing that included cytology, presence of HPV in cervical scrapings, and colposcopy. All patients underwent colposcopically-directed biopsies and endocervical curettage before treatment and at 6 months. The two study pathologists reviewed all biopsies obtained at the 6-month end point. Regression was defined as CIN 2 lesions reverting to normal or CIN 3 lesions converting to CIN 1 or normal. We defined persistent CIN 3 lesions as those that did not regress from the time of the initial biopsy to the 6-month end point. All patients with persistent CIN 3 or whose lesions progressed to CIN 3 at 6 months were taken off of the 2-year study and treated by conventional means.

### HPV analysis

We collected cervical scrapings from our patients for HPV analysis at enrolment, 3 and 6 months following the initial diagnostic CIN biopsy. Persistence and resolution of HPV infection was determined by comparing the analyses between the initial and 6-month time points. We defined HPV resolution as those with a presence of HPV DNA at initial entry and subsequently had undetectable levels of any HPV DNA at the 6-month end point. Furthermore, HPV persistence was defined as those with any HPV DNA both at baseline and 6 months. Although we detected mixed HPV types in a few patients, the majority of women had one HPV type at baseline that persisted throughout the 6 months.

Polymerase chain reaction (PCR) method was used to determine HPV genotype as described (Monk *et al*, 1994). DNA was first amplified with *β*-globin primers to confirm that the DNA extracted was intact and suitable for PCR. The DNA was then amplified with MY09/MY11 consensus primers that detect about 25 mucosal HPV types as well as type-specific primers for HPV 6, 16, and 18. The type-specific products were transferred to nylon membranes for hybridisation with ^32^P probes. MYO9/MY11 consensus products were assigned genotypes by RFLP digestion (Bernard *et al*, 1994) or if necessary sequenced. If no HPV DNA was detected with the primary primer sets, the DNA was then amplified with an additional consensus primer in the E1 gene (Gregoire *et al*, 1989) and the product sequenced.

### Risk factor analysis

We analysed the age, race, parity, smoking history, OCP use, marital status, lesion grade, sexual history, and HPV status on all our patients. We assessed the impact of various epidemiological risk factors on regression of CIN in all our patients using univariate (*χ*^2^) and multivariate (logistic regression) analyses performed with Statistical Analysis Systems. All *P*-values reported are two-tailed.

## RESULTS

From 1992 to 1996, 982 patients were screened for our study. We enrolled 124 women into our trial of whom 111 were eligible to be randomized to *β*-carotene or placebo. One hundred three patients had colposcopic persistence of CIN 2 or 3 one month after their initial biopsy. Ninety-three (90%) patients completed our ancillary study and underwent a 6-month cervical biopsy. The 10 excluded patients did not have a documented persistence of CIN 2 or 3 after initial biopsy. The median age of our patients was 29 years (range 19–55). The majority (59%) presented with CIN 3 lesions and 41% had CIN 2 disease. Approximately one-half (46/93) were Hispanic and the others were either Caucasian (*n*=45) or Asian (*n*=2). Fifty (54%) women were married or have been married in the past. Twenty-four (26%) women have completed high school or college. The median parity was one. Forty-two (45%) women had a history of OCP use. Twenty-eight (30%) patients were smokers and nine of these women were smoking more than one pack per day. Of the 75 women who completed the sexual history interview, 54 (72%) women had their first sexual intercourse at 18 years or younger (range: 11–30) with a median of two lifetime sexual partners (range: 1–25). At enrolment, HPV infection was detected in 84% (78 out of 93) of women and most patients had HPV types that were considered high risk for progressing to cancer. The HPV types at baseline included HPV type 16 (42%), type 33 (12%), type 18 (9%), type 31 (8%), type 52 (3%), type 53 (3%), type 35 (1%), type 39 (1%), and mixed/others (22%). Types 16, 18, and 33 were the most prevalent HPV types and 11 patients were infected with multiple HPV types.

We analysed the regression rates of CIN 2 and 3 with respect to various demographic and biological risk factors. The overall regression rate was 52% (48 out of 93), including 58% (22 out of 38) of CIN 2 lesions and 47% (26 out of 55) of CIN 3 lesions (*P*=0.31 for difference between grades). Interestingly, 80% (12 out of 15) of lesions without detectable levels of HPV regressed compared to only 46% (36 out of 78) of those infected with HPV (*P*=0.016). Of the 78 patients who initially tested positive for HPV, 24% (19 out of 78) subsequently developed undetectable levels of HPV. Of the patients who had a resolution of their HPV infection, 63% (12 out of 19) showed regression of their disease compared to a regression rate of only 41% (24 out of 59) in those who had a persistence of their viral infection. After the study, the population was divided into two groups based on the number of lifetime sexual partners: less than or equal to five and more than five. There was a significant inverse relationship (*P*=0.003) between number of sexual partners and probability of CIN regression (Table 1

**Table 1:** Univariate analyses

**Variable**	**Regression rate (%)**	***P*-value for difference**
*HPV status*		
Remained negative	80 (*n*=15)	
Resolved	63 (*n*=19)	
Persisted	41 (*n*=59)	0.004
		
*Number of sexual partners*		
⩽5	61	
>5	27	0.003
		
*Race*		
Hispanic	61	
White	40	0.05
		
*Age (years)*		
⩽30	44	
>30	61	0.11
		
*Parity*		
⩽2	46	
>2	64	0.11
		
*Smoking (ever)*		
Yes	39	
No	57	0.12
		
*Lesion grade*		
CIN 2	58	
CIN 3	47	0.31
		
*Age at first intercourse (years)*		
<18	48	
⩾18	57	0.48
		
*Use of oral contraceptives (ever)*		
Yes	48	
No	55	0.48
		
*Marital status*		
Single	49	
Ever-married	54	0.62

).

In univariate analyses, we found that absence of persistent HPV infection (*P*=0.005), less than or equal to five sexual partners (*P*=0.003), and Hispanic race (*P*=0.05) were all significant predictors for regression of high-grade dysplasia. Age, parity, smoking history, OCP use, marital status, lesion grade, and age at first intercourse were not significantly associated with CIN regression (Table 1). To further examine the variables identified as important in the univariate analyses, a multivariate logistic regression analysis was performed. We confirmed that the absence of HPV infection and less than or equal to five sexual partners remained as significant independent predictors for CIN regression (Table 2

**Table 2:** Multivariate analysis

**Variables**	**Odds ratio**	**95% CI**
*HPV infection*		
No	3.3	(1.3–8.6)
Yes	1	

*Number of lifetime sexual partners*		
⩽5	4.1	(1.4–11.5)
>5	1	

CI=confidence interval; HPV=human papillomavirus.

). After adjusting for HPV infection and number of sexual partners, Hispanic race was no longer a significant predictor of CIN regression. Inclusion of treatment group (*β*-carotene *vs* placebo) in the multivariate model did not change the estimates of risk of regression associated with HPV infection or number of sexual partners.

## DISCUSSION

Over the last 30 years, it has been widely accepted that CIN 1, 2, and 3 represent a continuum in the development of invasive cervical cancer (Kiviat, 1996). The biology of the various CIN grades vary, however, with the majority of CIN 1 lesions regressing without treatment (Nasiell *et al*, 1986; Montz *et al*, 1992; Flannelly *et al*, 1994), while CIN 2 and 3 lesions have a higher risk of persisting or progressing to invasive cancer. (Luthra *et al*, 1987; Murthy *et al*, 1996) Typically, most patients with CIN 2 or 3 lesions are promptly treated with ablation of the lesion or conisation of the cervix to prevent invasive disease. Consequently, studies on the natural history of untreated CIN 2 or 3 are limited (Nasiell *et al*, 1983; Luthra *et al*, 1987; Syrjanen *et al*, 1992; Flannelly *et al*, 1994). In addition, results from previous studies of the natural history of CIN are difficult to interpret because many studies depended on cytologic evaluation rather than histologic diagnosis to confirm either diagnosis or regression of disease. Furthermore, there often was a lack of uniform criteria in assigning a grade of dysplasia among the studies (Ostor, 1993; Holowaty *et al*, 1999). Lastly, repeat colposcopic examinations typically were not performed to confirm the persistence of CIN after the diagnostic biopsy. This lack of confirmation of persistent disease is important because many cervical lesions may regress after the initial diagnostic biopsy. All patients enrolled in our study had biopsy-proven and persistent CIN 2 or 3 lesions. Colposcopic examinations were performed 1 month after the initial biopsy to document disease persistence. Moreover, two pathologists agreed on the histologic diagnoses at the beginning and end of the study.

After all 93 patients were analysed in our study, we found that the regression rate of CIN 2 and 3 without conventional therapy was unexpectedly high at 52%. Regression included 58% of CIN 2 lesions and 47% of CIN 3 lesions. In contrast, Ostor's (1993) 40-year review of all reports that addressed the natural history of CIN found that the regression rates of CIN 2 and 3 averaged only 43 and 32%, respectively. Unfortunately, some of these studies were hampered by a failure to confirm persistent disease after initial biopsy and inconsistent methods of determining grade of CIN. As one of the few reports on biopsy-proven and persistent CIN 2 or 3 disease, we found that the regression rates of high-grade lesions are unexpectedly high. These findings might influence the decision to routinely manage all patients with high-grade dysplasia by ablation, LEEP, or cone biopsy, particularly if undetectable levels of HPV are present.

The presence of cervical HPV DNA often is associated with cytologic and histologic changes of CIN. Indeed, up to 90% of women with CIN are HPV DNA positive depending on the diagnostic method used (Kiviat *et al*, 1992; Schiffman, 1992). In our analysis, women who lacked detectable HPV by PCR following the biopsy had a significantly greater chance of CIN regression compared to those with persistent HPV infections. This finding is consistent with previous studies that have shown that persistent HPV infections enhance the development and persistence of squamous intraepithelial lesions (Karlsson *et al*, 1995; Romney *et al*, 1997; Ho *et al*, 1998). Furthermore, our patients who initially tested positive for HPV and subsequently developed undetectable levels of HPV had higher rates of CIN regression (63%) than patients with HPV persistence (41%). Previous data have also shown that the resolution of HPV infection reflects the natural regression patterns of squamous intraepithelial lesions (Nasiell *et al*, 1986; Daling *et al*, 1996). Since most HPV infections are transient (Rosenfeld *et al*, 1992; Hsing *et al*, 1994; Hinchliffe *et al*, 1995; Romney *et al*, 1997), the patients in our study with high-grade lesions, who had no detectable HPV in their cervical scrapings following the biopsy, may have had a resolving HPV infection. Thus, based on our findings and that of other investigations, it appears that the resolution of HPV infection is predictive of the spontaneous regression of high-grade lesions. This has strong implications for the role of antiviral therapies against HPV and subsequent regression of high-grade dysplasia. Unlike previous reports (Campion *et al*, 1986; Kataja *et al*, 1990), our study did not find a significant difference in regression rates between patients with high risk (16, 18, 31, 33, 35, 39, 45, 51, 52, 56, and 58) and other HPV types. However, we did find a lower rate of regression in patients infected with both low- and high-risk HPV types. Similarly, Ellerbrock *et al* (2000) also found that persistent HPV infection with or without type 16 or 18 was significantly associated with incident cervical lesions.

The role of HPV infection provides only a partial explanation for the high rate of spontaneous regression in our patient population. Sexual risk factors also have been recognised as causative determinants for CIN and cervical cancer. In our study, we found an inverse relationship between the number of sexual partners and CIN regression. Multivariate analyses of our study population showed that the effects of sexual risk factors persist even after controlling for HPV infections; suggesting that the presence of other sexually transmitted disease may also play a role in the natural history of CIN 2 or 3.

Our data propose that there may be selected patients with CIN who could be followed for short term in anticipation of spontaneous regression; this group includes those with no evidence of an HPV infection and low sexual risk factors. Indeed, the ASCUS-LSIL Triage Study reached this conclusion for patients with ASCUS Pap smears in whom HPV is not present (Solomon *et al*, 2001). Certainly, larger studies are needed to determine the safety and efficacy of conservative treatment of CIN 2 or 3; however, the short-term risk of progressing onto cancer in this population is very low (Holowaty *et al*, 1999). It is possible that the recommendation to observe women with ASCUS Pap smears who are negative for HPV may be applicable as short-term recommendations for women with established CIN, even when of high grade. Lastly, clinical trials are needed to determine the role of anti-HPV therapies including vaccines and immune modulators to prevent or eliminate HPV infection and potentially enhance the regression of CIN. With the high rate of spontaneous regression of CIN 2 and 3 in our patients, it may be safe and feasible to initiate a prospective, randomised trial to further investigate the natural regression of high-grade dysplasia.
